# Firearm Type and Number of People Killed in Publicly Targeted Fatal Mass Shooting Events

**DOI:** 10.1001/jamanetworkopen.2024.58085

**Published:** 2025-02-05

**Authors:** Leslie M. Barnard, Erin Wright-Kelly, Ashley Brooks-Russell, Marian E. Betz

**Affiliations:** 1 Firearm Injury Prevention Initiative, University of Colorado School of Medicine, Aurora; 2 Department of Epidemiology, University of Colorado School of Public Health, University of Colorado Anschutz Medical Campus, Aurora; 3 Injury and Violence Prevention Center, University of Colorado Anschutz Medical Campus, Aurora; 4 Department of Emergency Medicine, University of Colorado School of Medicine, Aurora

## Abstract

This cohort study examines mass shootings in the US to evaluate if assault weapons were associated with a higher number of injuries and deaths.

## Introduction

Mass shootings (MS) account for less than 1% of firearm deaths in the US, but the frequency has increased.^1^ Risk factors for MS perpetration include societal discrimination, contagion effects, firearm access, mental illness, and substance abuse.^2^ Previous geographically and analytically limited studies found MS with handguns had higher fatality rates than those with rifles,^3^ and following an age-based assault weapons (AWs) restriction there was a reduction in firearm violence from AWs.^4^ Another study found that the 1994 federal AWs ban was associated with fewer MS.^5^ To further investigate the association between type of firearm and lethality of MS, this study examined what firearms were present at publicly targeted fatal MS and determined if AWs were associated with a higher number of injuries or deaths.

## Methods

The retrospective cohort study was approved by the Colorado Multiple Institutional Review Board and used the Strengthening the Reporting of Observational Studies in Epidemiology (STROBE) reporting guideline. Data and definitions came from the Violence Project (eTable in Supplement 1). This study compared publicly targeted fatal MS (ie, an incident where more than 4 people are killed not including the shooter[s], in a public location, not attributable to any other crime) where an AW (any semiautomatic gun that can accept a detachable ammunition magazine that has 1 or more additional features considered useful in military applications) was present vs not by shooter and event characteristics. We used negative-binomial regression to model associations between firearm type and the number killed and the number nonfatally injured. Confounders (multiple firearms, firearm proficiency, familiarity with location, shooter age, and shooter sex) were identified a priori based on theory and empirical evidence.^2^ This analysis was limited to single shooter events. Significance was set at *P* < .05, and tests were 2-sided. Data were analyzed from August 1, 1966, to November 6, 2023, using SAS software version 9.4 (SAS Institute). Additional information can be found in the eMethods in Supplement 1.

## Results

From August 1, 1966, to November 6, 2023, there were 184 publicly targeted fatal MS (3.2 per year) with 1342 total deaths and 2084 nonfatal injuries. Multiple firearms were present in 96 events (52.2%) (mean 3.1 per event); handguns were the most common firearm type (145 MS [78.8%] involved a handgun) followed by 55 (29.2%) that involved an AW, but only 13 (7.1%) involved AWs exclusively. Incidents with AWs (vs without) were more likely to have multiple firearms (45 [81.8%] vs 51 [39.5%]) and shooters with no firearms experience (6 [11.1%] vs 38 [29.7%]) (Table 1).

**Table 1.** Firearm, Shooter, and Event Morbidity and Mortality of Fatal Mass Shootings From August 1, 1966, to November 6, 2023

Characteristic	Total mass shootings, No. (%) (N = 184)^a^	Incidents where an assault weapon was present, No. (%) (n = 55)	Incidents where an assault weapon was not present, No. (%) (n = 129)	*P *value^b^
Firearm characteristics				
Type of firearm^c^				
Assault weapon	55 (29.9)	55 (100)	NA	NA
Handgun	145 (78.8)	40 (72.7)	105 (81.4)	
Shotgun	39 (21.2)	13 (23.6)	26 (20.2)	
Rifle	35 (19.0)	8 (14.5)	27 (20.9)	
No. of firearms present				
1	88 (47.8)	10 (18.2)	78 (60.5)	<.001
2-4	86 (46.7)	38 (69.1)	48 (37.2)	
≥5	10 (5.4)	7 (12.7)	3 (2.3)	
Shooter characteristics				
Age, mean (SD), y	34.1 (12.6)	32.8 (12.4)	34.8 (12.7)	.87
Sex				
Male	179 (97.3)	52 (94.6)	127 (98.5)	.19
Female	3 (1.6)	1 (1.8)	2 (1.6)	
Other	2 (1.0)	2 (2.6)	0 (0.0)	
Shooter had an existing relationship with the shooting site	82 (44.6)	24 (43.6)	58 (45.0)	.87
Firearm proficiency				
No experience	44 (24.2)	6 (11.1)	38 (29.7)	.002
Some experience	56 (30.8)	18 (33.3)	38 (29.7)	
More experience	27 (14.8)	15 (27.8)	12 (9.4)	
Very experienced	55 (30.2)	15 (27.8)	31 (31.3)	
Event morbidity and mortality				
No. killed, median (IQR)	5 (4-7)	6 (5-10)	5 (4-7)	<.001
No. nonfatally injured, median (IQR)	3 (1-7)	6 (2-18)	2 (1-5)	<.001

Abbreviation: NA, not applicable.

^a^ The Violence Project defines mass shootings as “an incident in which 4 or more individuals were killed, not including the shooter(s); that occurred in a public location; and that was not attributable to any other underlying criminal activity.”

^b^ Two-sample *t* tests for continuous variables and χ^2^ for categorical variables.

^c^ The Violence Project defines firearm types as follows: handguns are firearms with a short barrel; shotguns are firearms with a long barrel (usually has a smooth bore); rifles are firearms with a long barrel with rifling; and an assault weapon is any semiautomatic gun that can accept a detachable ammunition magazine that has 1 or more additional features considered useful in military applications but unnecessary for sports or self-defense (ie, a folding, telescoping or thumbhole rifle stock). Assault weapons can be handguns, modified shotguns, or rifles. Categories are mutually exclusive, therefore, any type of firearm aligned to the database definition of assault weapon appears under assault weapon. Categories are not mutually exclusive because more than 1 type of firearm may be present at a single mass shooting event.

Incidents where an AW was present resulted in a mean of 0.72 (95% CI, 0.59-0.86) more people killed when controlling for confounding compared with events without an AW present. Incidents where an AW was present were also associated with the number injured after adjusting for confounding (0.27 [95% CI, 0.16-0.44]) (Table 2).

**Table 2.** Crude and Adjusted Estimates and 95% CI for Type of Firearm Present and Number Killed and Injured in Mass Shootings

Variable	Estimate (95% CI)	*P* value
**Assault weapon present and No. killed^a^**		
Crude	0.65 (0.54-0.78)	<.001
Adjusted^b^	0.72 (0.59-0.86)	<.001
**Assault weapon present and No. injured^a^**		
Crude	0.18 (0.12-0.29)	<.001
Adjusted^b^	0.27 (0.16-0.44)	.001

^a^ The Violence Project defines assault weapon as any semiautomatic gun that can accept a detachable ammunition magazine that has 1 or more additional features considered useful in military applications but unnecessary for sports or self-defense (ie, a folding, telescoping, or thumbhole rifle stock). Assault weapons can be handguns, modified shotguns, or rifles.

^b^ Adjusted for multiple firearms present, firearm proficiency, shooter familiarity with the location, shooter age, and sex.

## Discussion

We found most publicly targeted fatal MS involved multiple types of firearms and handguns were the most common type of firearm present. AWs being present during a publicly targeted mass shooting was associated with a slight increase in the number of injuries and deaths occurring during that incident.

There may be opportunities to intervene before a MS. Previous research found that plans to commit MS were disclosed before 47% of MS.^6^ Policies regulating firearm access show promise. Age-based AWs restrictions may be effective in reducing MS.^4^ Moreover, child access prevention laws, which require secure firearm storage to prevent child access, and extreme risk protection orders, which temporarily remove firearms from those who threaten violence, may be effective in preventing MS. Finally, secure storage in-home or out-of-home may be an effective strategy when risk for perpetration exists.

Limitations included the Violence Project definition of MS and AW. We used the type of firearms present as a proxy for use; this may have led to misclassification. Enhanced and systematic data collection is needed on MS in the US including border definitions of MS and the types of firearms used. Understanding contributing factors associated with MS can inform additional research, policies, and interventions.

## Notes

Supplement 1. **eMethods.** Search Strategy for Medline (Using PubMed) **eTable. **Variables and Definitions Used From the Violence Project Dataset

Supplement 2.
Data Sharing Statement
