# Supplementary material for: Firearm Type and Number of People Killed in Publicly Targeted Fatal Mass Shooting Events
Source: JAMA Netw Open. 2025 Feb 5;8(2):e2458085. doi: 10.1001/jamanetworkopen.2024.58085 (PMC11800013; doi:10.1001/jamanetworkopen.2024.58085)
Supplement: Supplement 1. — eMethods. Search Strategy for Medline (Using PubMed) eTable. Variables and Definitions Used From the Violence Project Dataset [file jamanetwopen-e2458085-s001.pdf]

Supplemental Online Content

Barnard LM, Wright-Kelly E, Brooks-Russell A, Betz ME. Firearm type and number of people killed in publicly targeted fatal mass shooting events. *JAMA Netw Open*. 2025;8(2):e2458085. doi:10.1001/jamanetworkopen.2024.58085

eMethods. Search strategy for Medline (using PubMed)
eTable. Variables and Definitions Used From the Violence Project Dataset

This supplemental material has been provided by the authors to give readers additional information about their work.

© 2025 Barnard LM et al. *JAMA Network Open*.

eMethods.

Dataset and Definitions

All data used in this analysis come from the Violence Project dataset. The Violence Project is a database supported by the National Institutes of Justice. The U.S. database has included incident characteristics – such as location, victims, firearms involved – and characteristics of mass shooters on all incidents since 1966 with a goal of identifying common traits of persons who engaged in mass shootings.

Currently there is no universally agreed upon definition of "Mass Shooting" or federal agency that systematically collects data on mass shootings in the U.S. Instead, several different organizations, such as the Violence Project, collect data using their own definitions. Depending on how broad or narrow these definitions are, these datasets produce different estimates of the number of mass shootings that occur in the U.S. The Violence Project uses a specific and narrow definition of "Mass shootings": an incident in which four or more were killed, not including the shooter(s) in a single event; the incident occurred in a public location; and the incident was not attributable to any other underlying criminal activity. We refer to these as "Publicly-Targeted Fatal Mass Shootings" (Supplement Table 1).

Incidents with fatalities often times have better media reporting; the Violence Project uses these media reports, police records, death records, among others to build its dataset. This includes crucial data on the number and type of firearms that are brought to these mass shooting incidents—a variable not included in other datasets that use a broader definition. Similar to the definition of "mass shootings" the definition for "assault weapon" varies and is often times contentious with critiques from many including the firearm community and policy makers. The Violence Project defines "assault weapon" as any semi-automatic gun that can accept a detachable ammunition magazine that has one or more additional features considered useful in military applications. This definition from the Violence Project is consistent with the Federal Assault Weapons Ban/ Public Safety and Recreational Firearms Use Protection Act of 1994 and was also used in this analysis (Table 1).

Statistical Analysis

This retrospective cohort study used data from 8/1/1966-11/6/2023 and compared incidents where an "assault weapon" was present versus not by firearms present, shooter, and

© 2025 Barnard LM et al. *JAMA Network Open*.

event characteristics. All data were assessed for missing and/or implausible data using frequency tables prior to formal analyses. This analysis was limited to single-shooter events.

First, we compared incidents where an "assault weapon" was present versus by firearms not present, shooter, and event characteristics, using two-sample t-tests or chi-squared tests. Next, we used negative-binomial regression to model the association between "assault weapon" present and number of people killed and non-fatally injured. A negative binomial regression was selected for use in over dispersed count data. Confounders (use of multiple firearms, firearm proficiency, shooter familiarity with the location, shooter age, and gender) were identified a-priori based on theory and prior empirical evidence; these are described further in Table 1. We ran two-sample t-tests for continuous variables and Chi square for categorical variables. All significance testing used an alpha level of 0.05. Results from regression are considered significant if the 95% confidence interval did not cross 0 and/or with and if the p-value is < 0.05).

eTable 1: Variables and Definitions Used From the Violence Project Dataset

Variable	Definition	Variable type
Publicly-targeted fatal mass shootings*	An incident in which four or more were killed, not including the shooter(s); that occurred in a public location; and that was not attributable to any other underlying criminal activity	N/A (case inclusion criteria from VP)
Type of firearm*	Assault Weapon: any semi-automatic gun that can accept a detachable ammunition magazine that has one or more additional features considered useful in military and criminal applications but unnecessary for sports or self-defense, such as a folding, telescoping or thumbhole rifle stock. This is consistent with the Federal Assault Weapons Ban of 1994. "Assault weapons" can be handguns, modified shotguns, or rifles.<br><br>Handguns: has a short barrel	Categorical (used to determine Assault Weapon Presence)

	Shot guns: has a long barrel and usually has a smooth bore<br><br>Rifle: long barrel with rifling, which puts spin on the bullet, increasing accuracy and distance<br><br>Categories are mutually exclusive, therefore any type of firearm aligned to the database definition of "assault weapon" appears under "assault weapon".	
**Exposure**		
Assault Weapon Presence	Fatal Mass Shooting Incidents where an "assault weapon" was present (at least one firearm that was brought to the scene was classified as an "assault weapon")	Categorical
**Outcomes**		
Number Injured *	Count of those non-fatally injured in the incident	Continuous
Number killed *	Count of those fatally injured in the incident	Continuous
**Confounding Variables**		
Use of multiple firearms	Where more than 1 firearm was present at the scene	Continuous
Firearm proficiency *	No experience, some experience, more experienced (held a permit or license, certifications or classes taken, more intensive practice before shooting), very experienced	Categorical
Shooter familiarity with the location *	The perpetrator has an existing relationship with the shooting site	Categorical
Shooter age *	Age at time of shooting	Continuous
Shooter gender *	Male/Female	Categorical

* Used the definition provided by the Violence Project database

© 2025 Barnard LM et al. *JAMA Network Open*.
