# Supplementary material for: Firearm Type and Number of People Killed in Publicly Targeted Fatal Mass Shooting Events
Source: JAMA Netw Open. 2025 Feb 5;8(2):e2458085. doi: 10.1001/jamanetworkopen.2024.58085 (PMC11800013; doi:10.1001/jamanetworkopen.2024.58085)
Supplement: Supplement 2. — Data Sharing Statement [file jamanetwopen-e2458085-s002.pdf]

Data Sharing Statement

Barnard. Firearm Type and Number of People Killed in Publicly Targeted Fatal Mass Shooting Events. *JAMA Netw Open*. Published February 05, 2025.
doi:10.1001/jamanetworkopen.2024.58085

Data
Data available: No

Additional Information
Explanation for why data not available: THis data is publically available
